# 653. Rapid Change in Microbiome Profiles Regardless of Diagnostic Method in a Post Hoc Comparative Analysis of Phase 3 Trials of Fecal Microbiota Spores, Live-brpk (formerly SER-109) for the Prevention of Recurrent *Clostridioides difficile* Infection (rCDI)

**DOI:** 10.1093/ofid/ofad500.716

**Published:** 2023-11-27

**Authors:** Anne J Gonzales-Luna, Kevin Litcofsky, Tim Straub, Dina Hot, Barbara McGovern, Brooke Hasson, Matthew Sims, Christopher Ford, Matthew Henn

**Affiliations:** Department of Pharmacy Practice and Translational Research, University of Houston College of Pharmacy, Houston, Texas, USA, Houston, TX; Seres Therapeutics, Inc., Cambridge, MA; Seres Therapeutics, Cambridge, MA, Cambridge, Massachusetts; Aimmune Therapeutics, a Nestlé Health Science company, Brisbane, CA, Brisbane, California; Seres Therapeutics, Inc., Cambridge, MA; Seres Therapeutics, Cambridge, MA, Cambridge, Massachusetts; Beaumont Health, Royal Oak, Michigan; Seres Therapeutics, Inc, Cambridge, MA; Seres Therapeutics, Cambridge, MA, Cambridge, Massachusetts

## Abstract

**Background:**

rCDI is characterized by diarrhea reemergence after symptom resolution on standard-of-care antibiotics and positive test for *C. difficile* toxin production or toxin gene presence. In two phase 3 trials with varied diagnostic testing methods, dosing with fecal microbiota spores, live-brpk (FMS; formerly SER-109) was associated with low rCDI rate ≤8 wks (ECOSPOR III: FMS, 12% vs placebo, 40%; ECOSPOR IV: FMS, 9%). In ECOSPOR IV, recurrence rates with FMS ≤8 wks were similar by diagnostic method (10.4% [95% CI, 6.5%–15.6%] in patients [pts] toxin-positive by EIA with/without PCR vs 4.3% [95% CI, 0.9%–12.2%] in pts with toxin gene detection by PCR only [PCR+]). We evaluated (post hoc) if pt microbiome profiles differed by diagnostic method at study entry.

**Methods:**

Baseline (pre-dose) stool samples were obtained from pts in *a*) ECOSPOR III, a placebo-controlled trial in pts with ≥2 prior rCDI episodes and diagnosis by toxin EIA (TOX+) and *b*) ECOSPOR IV, an open-label trial in pts with ≥1 prior rCDI and diagnosis by TOX+ with/without PCR or PCR+. Pts had ≥3 unformed stools/day for 2 consecutive days and symptom resolution on antibiotics before enrolling. Samples were analyzed for alpha diversity and engraftment of dose species not present at baseline using whole metagenomic sequencing. Concentrations of primary bile acids (1°BA), which cause *C. difficile* spore germination, were measured by targeted LC–MS panel. Statistical analyses in FMS-treated pts compared Shannon diversity, engraftment, and 1°BA concentration changes from paired baseline–Week 1 samples between subgroups.

**Results:**

Baseline Shannon diversity was similarly low across TOX+ and PCR+ subgroups (*P*>0.05); engraftment magnitude after FMS was comparable across trials (*P*>0.05) (**Fig 1**). Baseline 1°BA concentrations were comparable across trials (*P*>0.05) and subgroups (*P*>0.05) and decreased by Week 1 after FMS in TOX+ (*P*< 0.001) and PCR+ (*P*< 0.001) subgroups (**Fig 2**).

**Figure:**
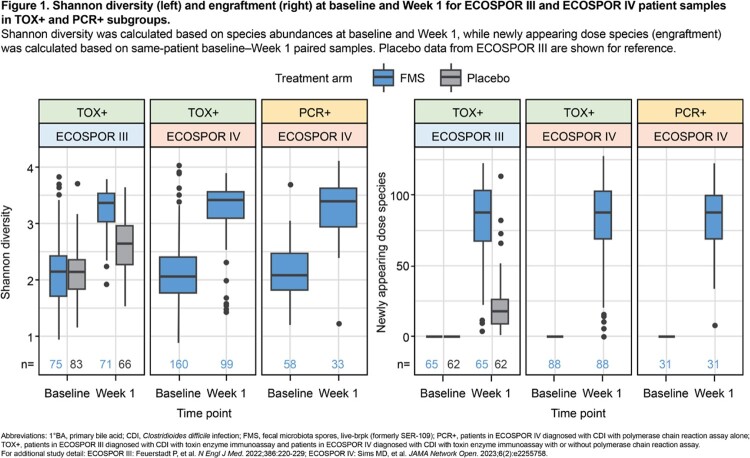

**Figure:**
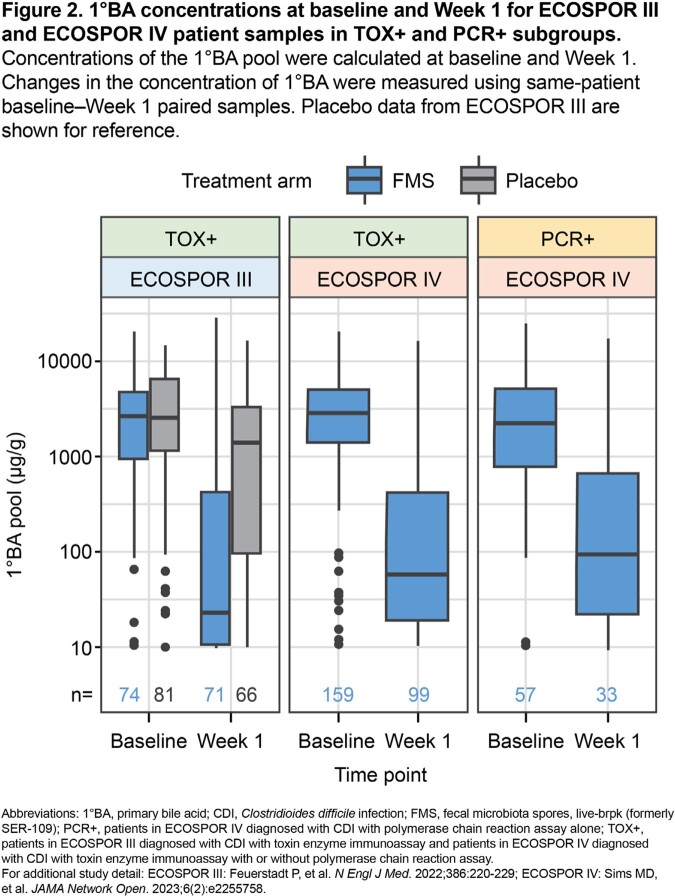

**Conclusion:**

Regardless of diagnostic method, microbiota diversity appeared similarly disrupted at baseline and improved rapidly by 1 wk after FMS. Engraftment magnitude and 1°BA concentration changes appeared similar across diagnostic subgroups. These data further support the low recurrence rates shown in trials of FMS.

**Disclosures:**

**Anne J. Gonzales-Luna, PharmD, BCIDP**, Cidara Therapeutics: Grant/Research Support|Ferring Pharmaceuticals: Personal Fees|Paratek Pharmaceuticals: Grant/Research Support|Seres Therapeutics: Grant/Research Support **Kevin Litcofsky, PhD**, Seres Therapeutics: inventor on patents assigned to Seres Therapeutics|Seres Therapeutics: Employment|Seres Therapeutics: Stocks/Bonds **Tim Straub, MS**, Seres Therapeutics: Employee|Seres Therapeutics: Stocks/Bonds **Dina Hot, PhD**, Aimmune Therapeutics: Employee **Barbara McGovern, MD**, Seres Therapeutics: Stocks/Bonds **Brooke Hasson, PhD**, Sage Therapeutics: Stocks/Bonds|Seres Therapeutics: Stocks/Bonds **Matthew Sims, MD, PhD**, Adaptive Phage Therapeutics: Grant/Research Support|Applied Biocode: Advisor/Consultant|Biotest AG: Grant/Research Support|ContraFect: Grant/Research Support|Finch: Grant/Research Support|Janssen: Grant/Research Support|Leonard-Meron Biosciences: Grant/Research Support|Merck and Co: Grant/Research Support|Novozyme: Grant/Research Support|OpGen: Advisor/Consultant|OpGen: Grant/Research Support|Pfizer: Grant/Research Support|Prenosis: Advisor/Consultant|Prenosis: Grant/Research Support|QIAGEN: Grant/Research Support|Seres: Grant/Research Support|Summit Therapeutics: Grant/Research Support **Christopher Ford, PhD**, Seres Therapeutics: Employee|Seres Therapeutics: Stocks/Bonds **Matthew Henn, PhD**, Seres Therapeutics: Employee|Seres Therapeutics: Stocks/Bonds

